# Controllable Synthesis of Na_3_V_2_(PO_4_)_3_/C Nanofibers as Cathode Material for Sodium-Ion Batteries by Electrostatic Spinning

**DOI:** 10.3389/fchem.2018.00617

**Published:** 2018-12-10

**Authors:** Ling Wu, Yueying Hao, Shaonan Shi, Xiaoping Zhang, Huacheng Li, Yulei Sui, Liu Yang, Shengkui Zhong

**Affiliations:** ^1^School of Iron and Steel, Soochow University, Suzhou, China; ^2^Citic Dameng Mining Industries Limited, Chongzuo, China

**Keywords:** sodium-ion batteries, cathode materials, Na_3_V_2_(PO_4_)_3_, electrospinning, nanofibers

## Abstract

Na_3_V_2_(PO_4_)_3_/C nanofibers are prepared by a pre-reduction assisted electrospinning method. In order to maintain the perfect fibrous architecture of the Na_3_V_2_(PO_4_)_3_/C samples after calcining, a series of heat treatment parameters are studied in detail. It is found that the heat treatment process shows important influence on the morphology and electrochemical performance of Na_3_V_2_(PO_4_)_3_/C composite nanofibers. Under the calcining conditions of 800°C for 10 h with a heating rate of 2.5°C min^−1^, the well-crystallized uniform Na_3_V_2_(PO_4_)_3_/C nanofibers with excellent electrochemical performances are successfully obtained. The initial discharge specific capacities of the nanofibers at 0.05, 1, and 10C are 114.0, 106.0, and 77.9 mAh g^−1^, respectively. The capacity retention still remains 97.0% after 100 cycles at 0.05C. This smooth, uniform, and continuous Na_3_V_2_(PO_4_)_3_/C composite nanofibers prepared by simple electrospinning method, is expected to be a superior cathode material for sodium-ion batteries.

## Introduction

As an alternative strategy to lithium-ion batteries (LIBs), sodium-ion batteries (SIBs) recently have been paid increasing attention due to the low cost and the abundant reserves of sodium resource in the earth (Chen et al., 2018; Wu et al., 2018a,b; Ge et al., 2019). Nevertheless, comparing with Li-ion, Na-ion has larger ionic radius (1.02 Å), and heavier atomic weight, which is not conducive to the ion diffusion during the inserting/extracting processes. Thus, those compounds which have open framework are more suitable for the transmission of Na-ions. Among the cathode materials of SIBs, Na_3_V_2_(PO_4_)_3_ is concerned widely due to its NASICON structure. The theoretical specific capacity of Na_3_V_2_(PO_4_)_3_ is 117 mAh g^−1^ (de-intercalating 2 Na^+^), and its average operating voltage is as high as 3.4 V. Most importantly, the NASICON structure can provides faster channels for the insertion/extraction of Na-ions, making Na_3_V_2_(PO_4_)_3_ has relatively higher ionic conductivity than many other polyanionic compounds (Nanjundaswamy et al., 1996; Song et al., 2014a,b). However, the absence of electronic delocalization through direct -M-O-M-links results in a poor intrinsic electronic conductivity, and to some degree, the Na-ion diffusion is still limited by its large radius and heavy weight. Thus, the electronic/ionic conductivities of Na_3_V_2_(PO_4_)_3_ further need to be improved (Jamesh and Prakash, 2008; Palomares et al., 2013; Kim et al., 2018). In order to increase the conductivity of cathode material, conductive agent coating (Prosini et al., 2001; Si et al., 2014; Ali et al., 2016; Chu and Yue, 2016), and particle size refining (Klee et al., 2016b; Wei et al., 2017; Zhang et al., 2017; Zheng et al., 2017) are usually used for modification. Compared with the solid-state method (Jian et al., 2012; Klee et al., 2016a), sol-gel method (Lim et al., 2012; Böckenfeld and Balducci, 2014), or solution evaporation method (Zheng et al., 2017), electrospinning is an effective way to achieve both of these goals. The Na_3_V_2_(PO_4_)_3_ nanofibers prepared by electrospinning can weave a three-dimensional (3D) conductive network which is beneficial for the fast transmission of electrons and sodium-ions (Chen et al., 2012, 2015; Zhong et al., 2016). Therefore, nano-sized Na_3_V_2_(PO_4_)_3_ fibers wrapped with amorphous carbon are expected to exhibit better electrochemical performances.

Recently years, one-dimensional Na_3_V_2_(PO_4_)_3_/C composites have been synthesized by electrospinning method (Kajiyama et al., 2014; Liu et al., 2014; Li et al., 2015a,b). Nanofibers synthesized by Kajiyama et al. (2014) showed large size differences after heat treatment, giving rise to an unsatisfactory cycle stability. (Liu et al., 2014); Li et al. (2015a) reports that Na_3_V_2_(PO_4_)_3_/C nanofibers obtained at 800°C tends to have a more uniform nanorod shape, but the rate performance of the nanofibers still needs to be enhanced. Li et al. (2015a) synthesized the Na_3_V_2_(PO_4_)_3_/C with budding willow branches shape by electrospinning method as well, yielding a discharge capacity of 116.2 mAh g^−1^ at 0.05 C rate, but its rate performance was not investigated further in the paper.

Lately, the smooth and uniform nano-sized Na_3_V_2_(PO_4_)_3_/C composite fibers are successfully prepared by our group through a pre-reduction assisted electrospinning method (Wu et al., 2018c). The synthesized Na_3_V_2_(PO_4_)_3_/C nanofibers present significantly improved electrochemical performances. It is generally believed that the particle size, morphology, and structure can greatly affect the material electrochemical properties. While heat treatment conditions (such as temperature, duration, and heating rate) have direct effects on the morphology, structure, and particle size of nanofiber materials (Chen et al., 2015; Xu et al., 2015; Jing et al., 2016). However, the effect of heat treatment parameters on the properties of the Na_3_V_2_(PO_4_)_3_/C wires after electrospinning has not been studied yet. Therefore, a series of electrospun Na_3_V_2_(PO_4_)_3_/C nanofibers under different heating conditions were studied in detail in this paper. As the heat treatment parameters are adjusted, the morphologies of the Na_3_V_2_(PO_4_)_3_/C nanofibers represent a series of significant and regular changes. The effect of morphology on its electrochemical performance is further discussed in particular below.

## Experimental

### Synthesis of Materials

Na_3_V_2_(PO_4_)_3_/C nanofibers were synthesized by the following procedures. (1) 0.12 M solution (30 mL) was prepared using H_2_C_2_O_4_·2H_2_O and deionized water and (2) NH_4_VO_3_ and NaH_2_PO_4_ (V: Na = 2:3, molar ratio) were added into the above solution, and a green solution (Solution I) was got after mixing at 70°C for 2 h. (3) Polyvinylpyrrolidone (PVP K90, MW = 1,300,000) was added into the deionized water. After stirring for 2 h at room temperature, 1.5 g mL^−1^ PVP solution (Solution II, 30 mL) was obtained. (4) Solution I was mixed with solution II under stirring, and then the final spinning solution (Solution III) was obtained after 2 h. (5) Solution III was pumped into an injector with a stainless steel needle pipe (inside diameter: 0.6 mm). In the spinning process, the distance, and voltage between the collector (Al-foil) and needle tip is 25 cm and 12 kV, respectively. The injection speed of spinning is 0.05 mL min^−1^. Then the nanofibers of precursor were collected and dried at 120°C in oven for 12 h. (6) The nanofibers of precursor were calcined at 750–900°C in Ar atmosphere for 6–12 h with the different heating rate of 1–5°C min^−1^. And a series of Na_3_V_2_(PO_4_)_3_/C composite nanofibers were obtained after cooling to ambient temperature.

### Characterization

The phase and crystal structure of Na_3_V_2_(PO_4_)_3_/C samples were characterized by X-ray diffraction (XRD, Rigaku ultima VI). The morphology of Na_3_V_2_(PO_4_)_3_/C nanofibers was observed by scanning electron microscopy (SEM, Hitachi-SU5000). The amount of residual carbon of samples was measured by a C-S analyzer (Eltar, Germany).

### Electrochemical Measurements

The positive electrode plate was prepared with the Na_3_V_2_(PO_4_)_3_/C samples, acetylene black and PVDF (8:1:1, weight ratio) by using N-methylpyrrolidone (NMP) solvent with an Al-foil as current collector. The electrode loading density is about 2.5 mg cm^−2^. The button batteries (CR2025) were assembled in an Ar-filled glove box. A glass fiber membrane (Whatman, GF/A) and a metallic Na-foil were used as the separator and negative electrode, respectively. The NaClO_4_ (1 M) solution in propylene carbonate (PC) and fluoroethylene carbonate (FEC) (1:0.05 in volume) was used as the electrolyte. The electrochemical performances of cells were tested on a LAND BT2013A battery tester at ambient temperature. The cells were charged/discharged at 0.05–10 C rates (1C = 118 mAh g^−1^) between the potentials (vs. Na/Na^+^) of 2.5 and 4.2 V. The electrochemical impedance spectroscopy (EIS) was measured by a CHI 660D workstation with the amplitude of 5 mV and the frequency range of 0.01–100 kHz.

## Results and Discussion

**Figure 1:**
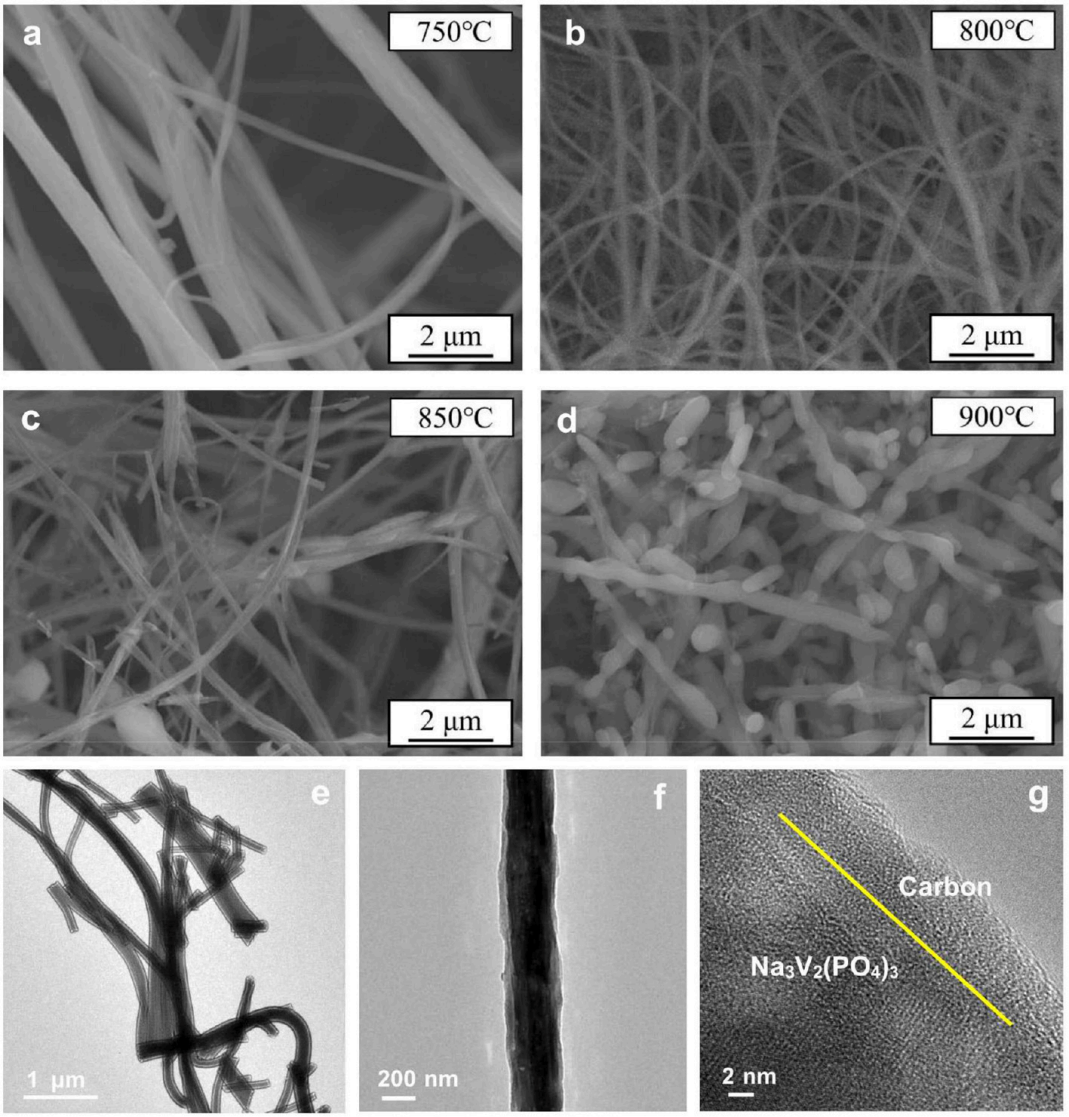
SEM images of Na_3_V_2_(PO_4_)_3_/C synthesized at different temperatures **(a–d)**. TEM images of Na_3_V_2_(PO_4_)_3_/C synthesized at 800°C **(e–g)**.

The main challenge of obtaining excellent Na_3_V_2_(PO_4_)_3_/C nanofibers is the structure stability during the heat treatment process, which requires to prevent the damage to the carbon layer and the fiber morphology. (Figures 1a–d) shows the SEM images of the Na_3_V_2_(PO_4_)_3_/C nanofibers prepared at 750–900°C. As shown, the samples calcined at 750 and 800°C exhibit continuous fibrous morphology. Furthermore, the diameter of nanofibers synthesized at 800°C is finer and more mean. When the heating temperature rises to 850°C, the nanofibers are broken. The surface of the sample prepared at 900°C can be unable to withstand the thermal stress so that the nanowires are completely disconnected and tend to grow into larger particles. Thus, the synthesis temperature exhibits a significant effect on the morphology of Na_3_V_2_(PO_4_)_3_/C nanofibers. And if the calcining temperature is not higher than 800°C, the filamentous morphology of nanowires is more likely to be preserved. In addition, the TEM images in (Figures 1e–g) prove that the surface of nanofibers is uniformly and smoothly coated by amorphous carbon layer with a thickness of several nanometers.

**Figure 2:**
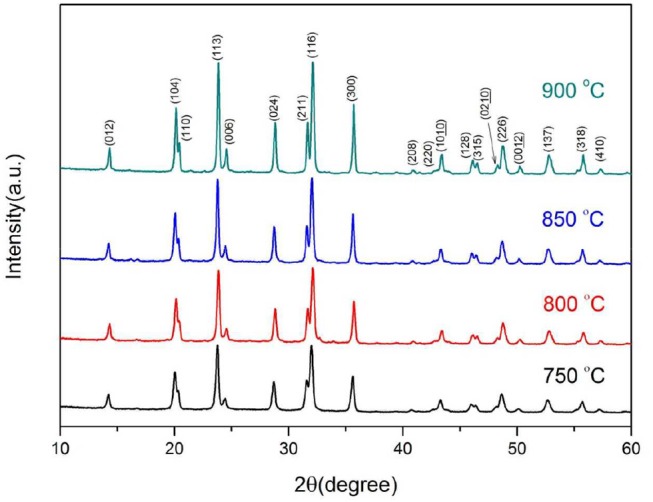
XRD patterns of Na_3_V_2_(PO_4_)_3_/C synthesized at different temperatures.

Figure 2 represents the XRD patterns of the samples synthesized at various temperatures. As shown, the diffraction peaks of all the samples are sharp and well-defined. All the samples can be fully indexed to Na_3_V_2_(PO_4_)_3_ phase (space group of *R-3C*) (Zatovsky, 2010), and no impurity phases are detected. The C-S analysis shows that the residual carbon contents of the samples calcined at 750–900°C are 12.1, 11.7, 10.4, and 9.8%, respectively. The residual carbon content decreases as the calcining temperature increases. However, there are no obvious diffraction peaks of carbon can be observed, indicating the carbon is amorphous. The lattice constants of samples are listed in Table 1. As shown, both *a* and *c* increase with the synthesis temperature, indicating that the calcining process strengthened the crystallization of the samples.

**Table 1:** The lattice parameters of Na_3_V_2_(PO_4_)_3_/C synthesized at different temperatures.

**Samples (^**°**^C)**	**Lattice parameters**		***V*/Å^**3**^**
	***a*/Å**	***c*/Å**	
750	8.7133	21.7996	1433.32
800	8.7272	21.8035	1438.16
850	8.7365	21.8092	1441.60
900	8.7436	21.8122	1444.14

**Figure 3:**
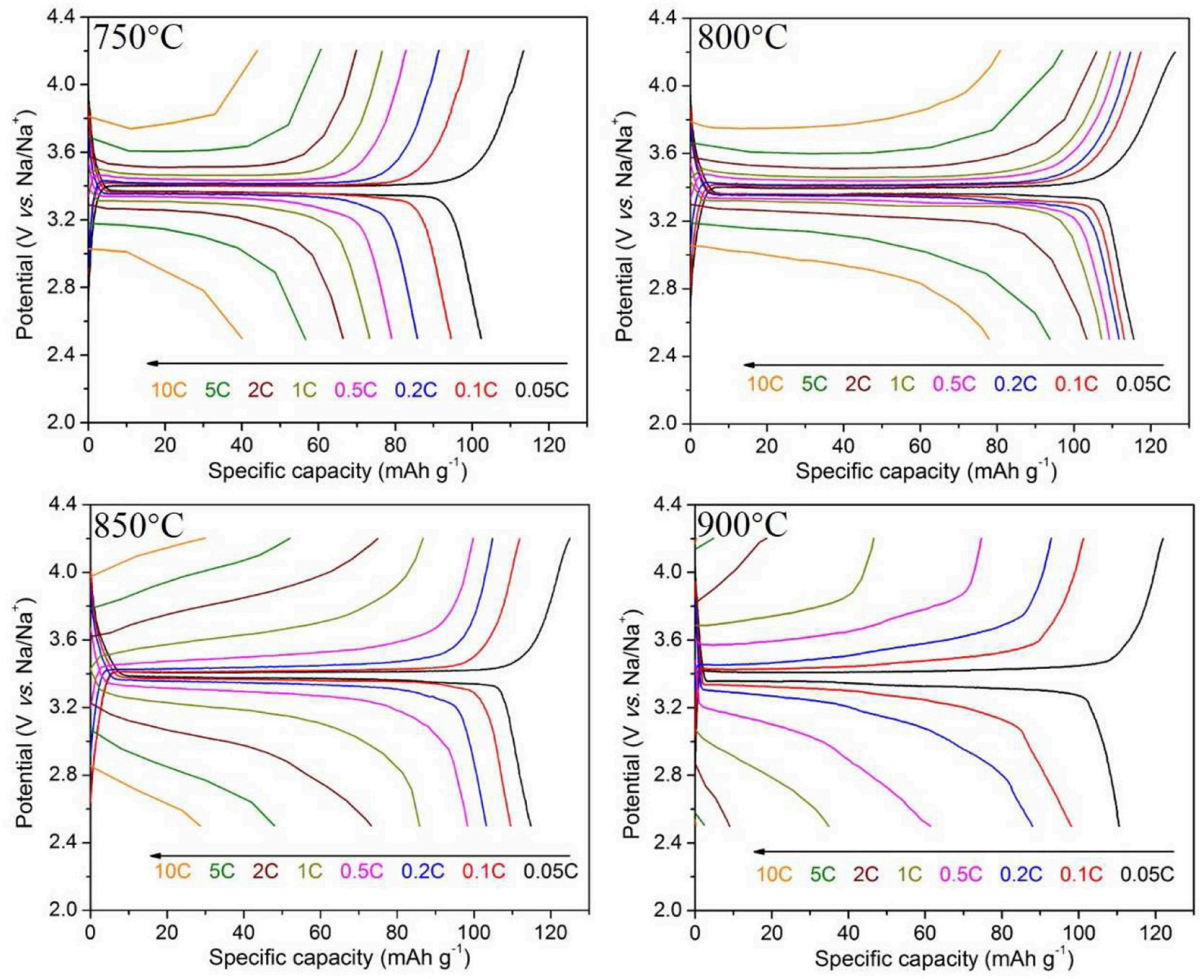
Charge/discharge curves of Na_3_V_2_(PO_4_)_3_/C synthesized at different temperatures.

The charge-discharge curves at 0.05–10 C rates of Na_3_V_2_(PO_4_)_3_/C samples synthesized at different temperatures are shown in Figure 3. As seen, all the samples show an obvious charge and discharge platform near 3.4 V. The discharge specific capacities at various rates increases first and then decreases with the heat temperature rising. When the calcining temperature reaches 800°C, the material possesses the optimal comprehensive performances with the highest specific capacities and best rate capability. It exhibits a first discharge specific capacity of 114.0 mAh g^−1^ at 0.05 C rate, close to the theoretical capacity, and still maintains a capacity of 77.9 mAh g^−1^ at 10 C.

Although all samples are well-crystallized according to the XRD results, there are large differences in electrochemical properties among these samples. The reasons can be ascribed to the various morphologies of samples obtained at different temperatures. In particular, the core-shell Na_3_V_2_(PO_4_)_3_/C nanofibers synthesized at 800°C exhibit continuous, smooth, and uniform filamentous architecture (Figures 1e–g). The Na_3_V_2_(PO_4_)_3_ fibers are wrapped with a carbon layer with a suitable thickness. In contrast, the nanofibers obtained at 750°C are non-uniform and the average diameter is thicker, and samples at the region of 850–900°C evenly cannot maintain fibrous structure. Thus, there are significant interconnections between the morphological characteristics of Na_3_V_2_(PO_4_)_3_/C nanowires and their electrochemical properties. In order to obtain better comprehensive electrochemical performances for the materials, the core-shell fibers should be continuous, smooth, and uniform and coated by proper carbon layer. Therefore, adjusting the electrospinning preparation conditions (adding oxalic acid and optimizing the heat treatment condition) can lead to excellent electrochemical properties of the material.

**Figure 4:**
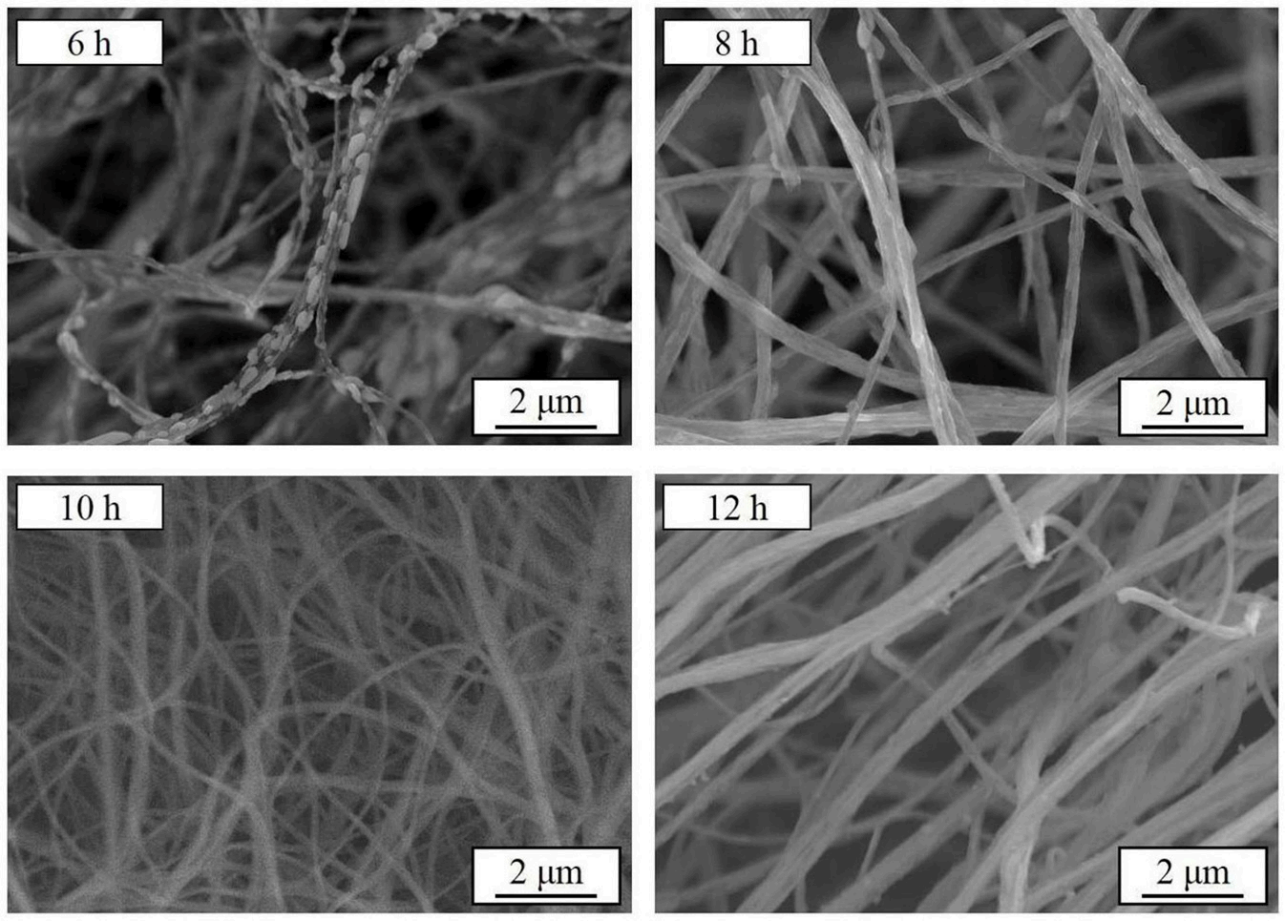
SEM images of Na_3_V_2_(PO_4_)_3_/C synthesized at different calcining times.

**Figure 5:**
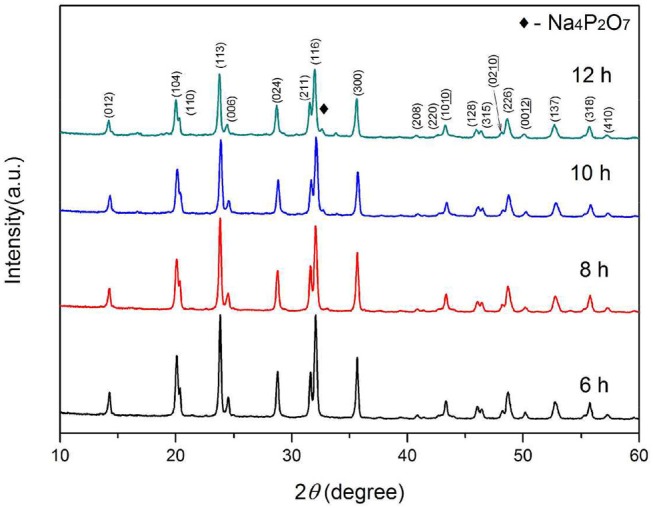
XRD patterns of Na_3_V_2_(PO_4_)_3_/C synthesized at different calcining times.

Further, the samples are synthesized at a heating rate of 2.5°C min^−1^ at 800°C for 6–12 h, respectively. Figure 4 presents the SEM images of the as-synthesized samples. After 6 and 8 h heat treatment, the originally smooth nanowires precursor become finer branches with crystalline particles on the surface. As the calcining time is prolonged, the branch-like crystalline particles are re-melted into the nanowires. The surface of the fibrous material becomes smooth and its diameter increases. The XRD patterns of the Na_3_V_2_(PO_4_)_3_/C materials obtained under the above conditions are shown in Figure 5. The crystallized Na_3_V_2_(PO_4_)_3_ without obvious diffraction peak of carbon can be obtained. The presence of Na_3_V_2_(PO_4_)_3_ grains on the surface of the nanowires responds to a higher intensity diffraction peak in the XRD patterns. As the calcining time is prolonged, the intensity of the diffraction peak slightly decreases. When the calcining time reaches 12 h, the Na_4_P_2_O_7_ (JCPDS 10-1087) heterogeneous phase appears. The carbon contents of the samples calcined for 6, 8, 10, and 12 h are 12.7, 12.2, 11.9, and 11.5%, respectively.

**Figure 6:**
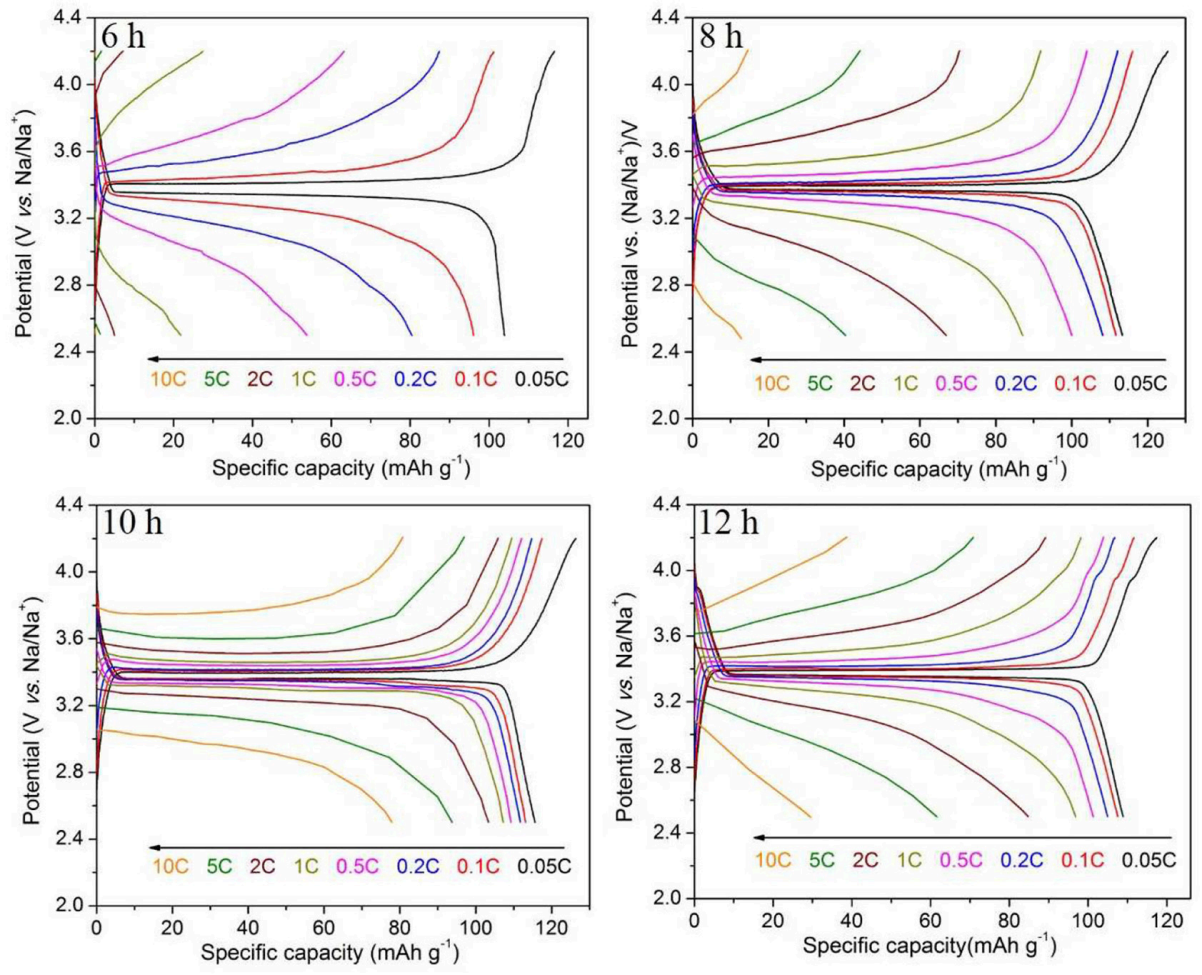
Charge/discharge curves of Na_3_V_2_(PO_4_)_3_/C synthesized at different calcining times.

The charge and discharge curves of the samples with heat treatment time of 6–12 h are shown in Figure 6. It is obvious that the sample synthesized for 10 h shows the excellent properties. The sample synthesized for 8 h with grains on the surface has a first discharge specific capacity of 113.4 mAh g^−1^ at 0.05 C, but only 13.0 mAh g^−1^ at 10 C. The capacity decay is more pronounced in the charge and discharge test of the sample synthesized for 6 h. These indicate that the electrochemical performances of Na_3_V_2_(PO_4_)_3_/C grains are much worse than the intact Na_3_V_2_(PO_4_)_3_/C nanofibers, which is consistent with the above results. Compared with the granular samples, the continuous, smooth, and uniform nanofibers evenly coated by a carbon layer can build up a perfect network with high electronic conductivity, which will greatly enhance the rate performances of Na_3_V_2_(PO_4_)_3_, especially at high current rates. An additional small platform around 3.9 V appears in the charging curve of the sample synthesized for 12 h, corresponding to the appearance of the heterogeneous phase in the XRD pattern. Thus, as the calcining time extended, the nanofibers gradually become more smooth, and continuous, and exhibiting a better rate performance. However, if the calcining time is too long, heterogeneous phase appears in the synthesized samples would lead to a decreased electrochemical property.

**Figure 7:**
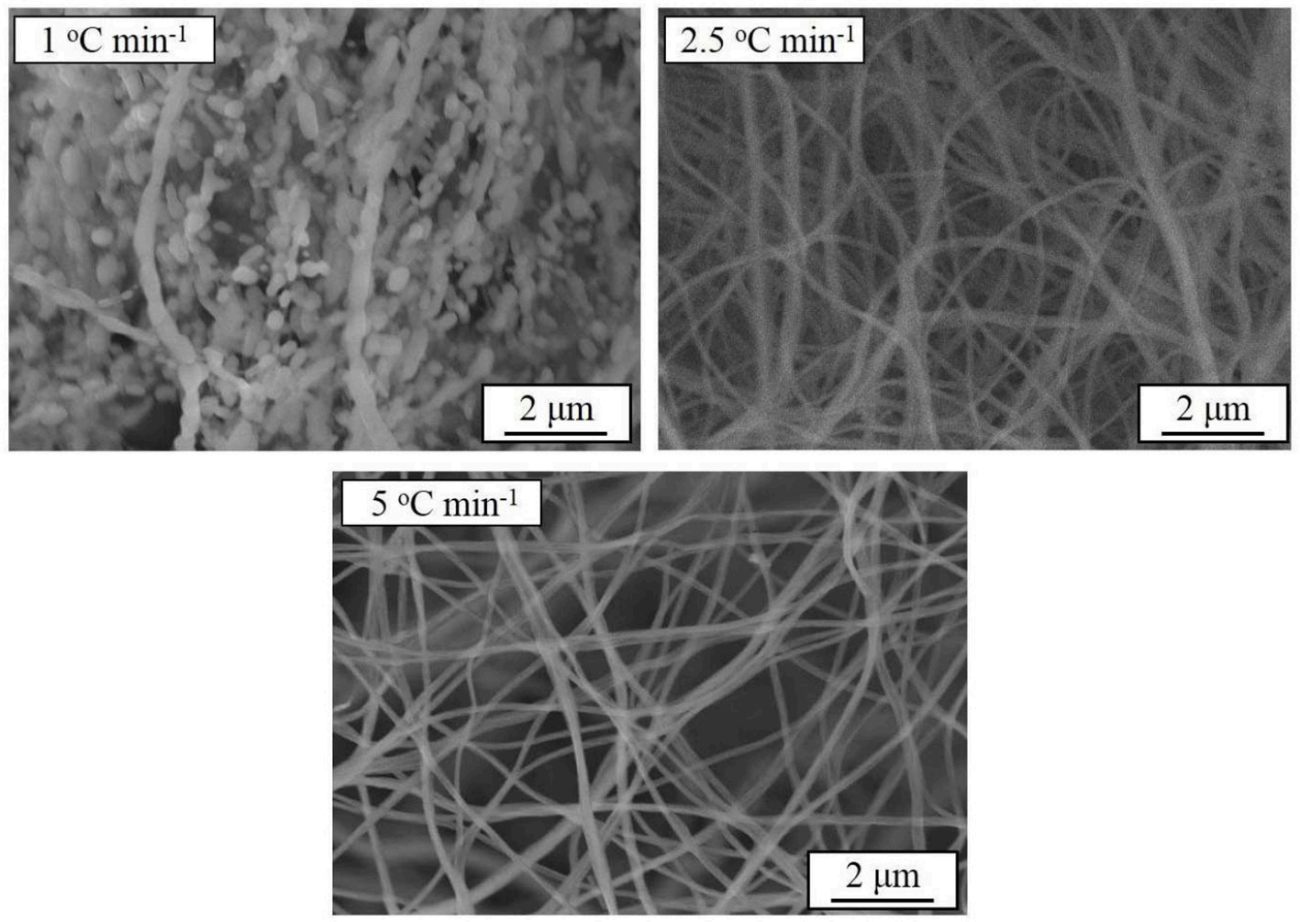
SEM images of Na_3_V_2_(PO_4_)_3_/C synthesized at different heating rates.

Based on the above results, another batch of samples are synthesized at 800°C for 10 h with different heating rates. The SEM images of the samples prepared at different heating rates are shown in Figure 7 At the heating rate of 1°C min^−1^, the filament structure of the precursor is completely destroyed due to the excessive warm-up time, which is similar to the sample morphology at 900°C in Figure 1. In order to keep the nanometer filament of the material, the heating rate is increased to 2.5 and 5°C min^−1^. Obviously, the faster the heating rate is, the smaller the diameter of the wire gets.

**Figure 8:**
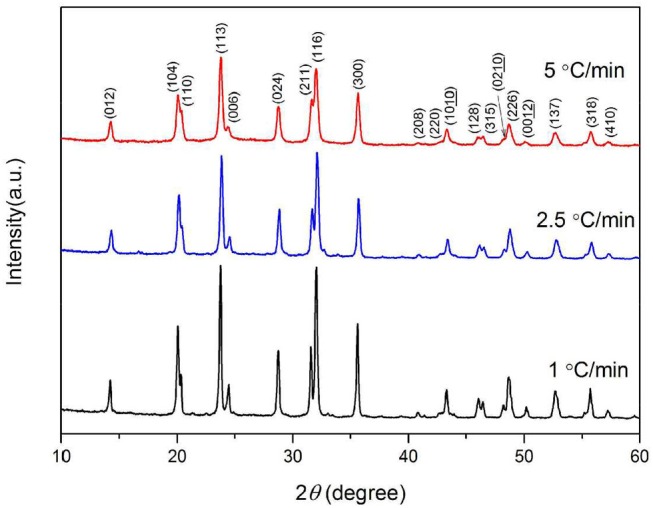
XRD patterns of Na_3_V_2_(PO_4_)_3_/C synthesized at different heating rates.

The XRD patterns of the Na_3_V_2_(PO_4_)_3_/C materials obtained under the above conditions are shown in Figure 8. It shows that the slower of the calcining speed, the stronger of the samples' diffraction peaks. In the XRD pattern of 5°C min^−1^ sample, the intensity of diffraction peak is lower, and the half width is larger which may be the result of insufficient diffusion of elements during the process of heat treatment under a fast heating rate.

**Figure 9:**
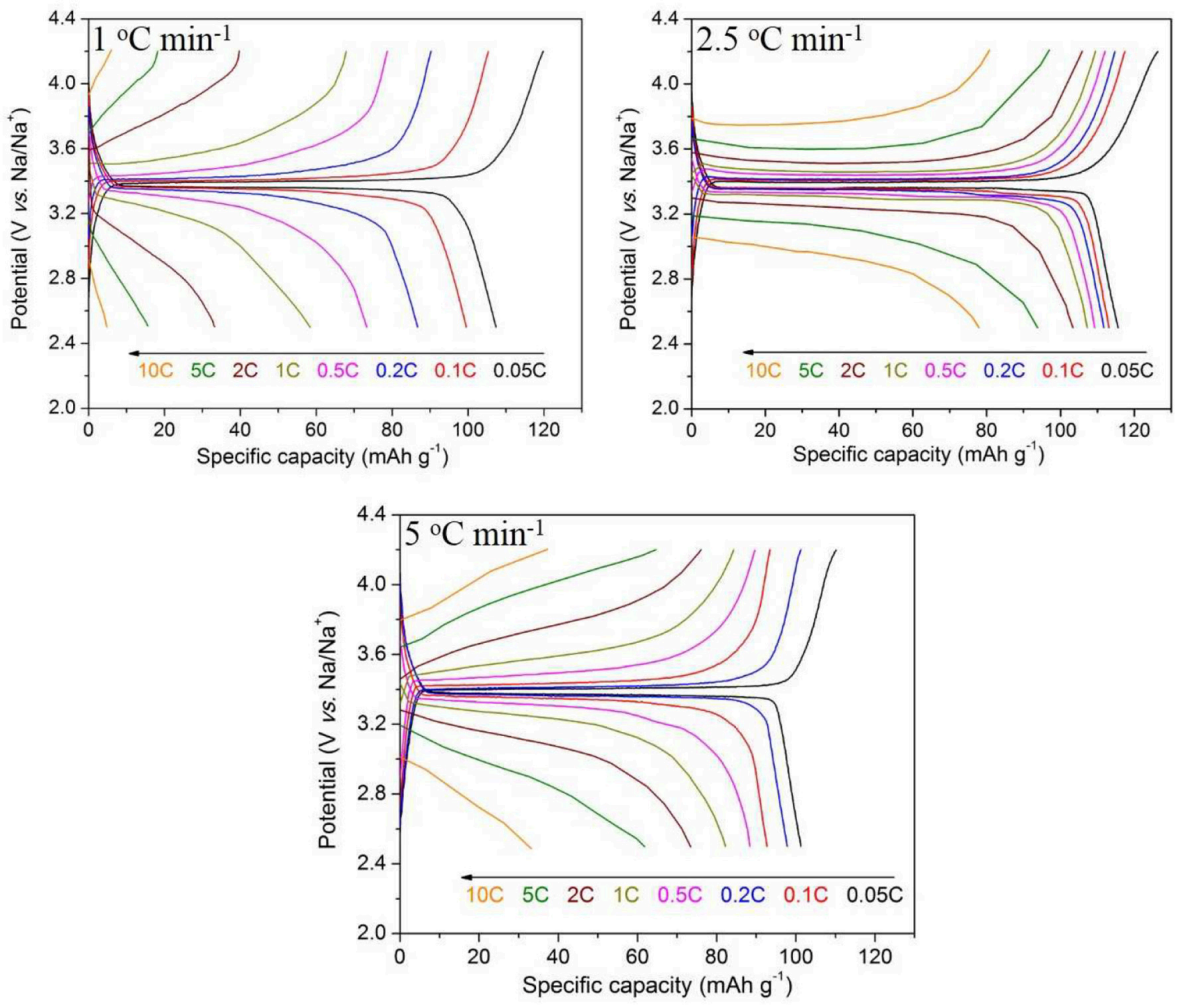
Charge/discharge curves of Na_3_V_2_(PO_4_)_3_/C synthesized at different heating rates.

As shown in Figure 9, the electrochemical performance of the sample heated at 1°C min^−1^ is even worse than that of the 900°C sample because of the most loss of filamentous morphology. The initial discharge capacity of the sample heated at the rate of 5°C min^−1^ is only 108.8 mAh g^−1^ at 0.05 C, and its capacity decay is obvious at low current density. This may be due to the unsatisfied crystallization of the Na_3_V_2_(PO_4_)_3_.

**Figure 10:**
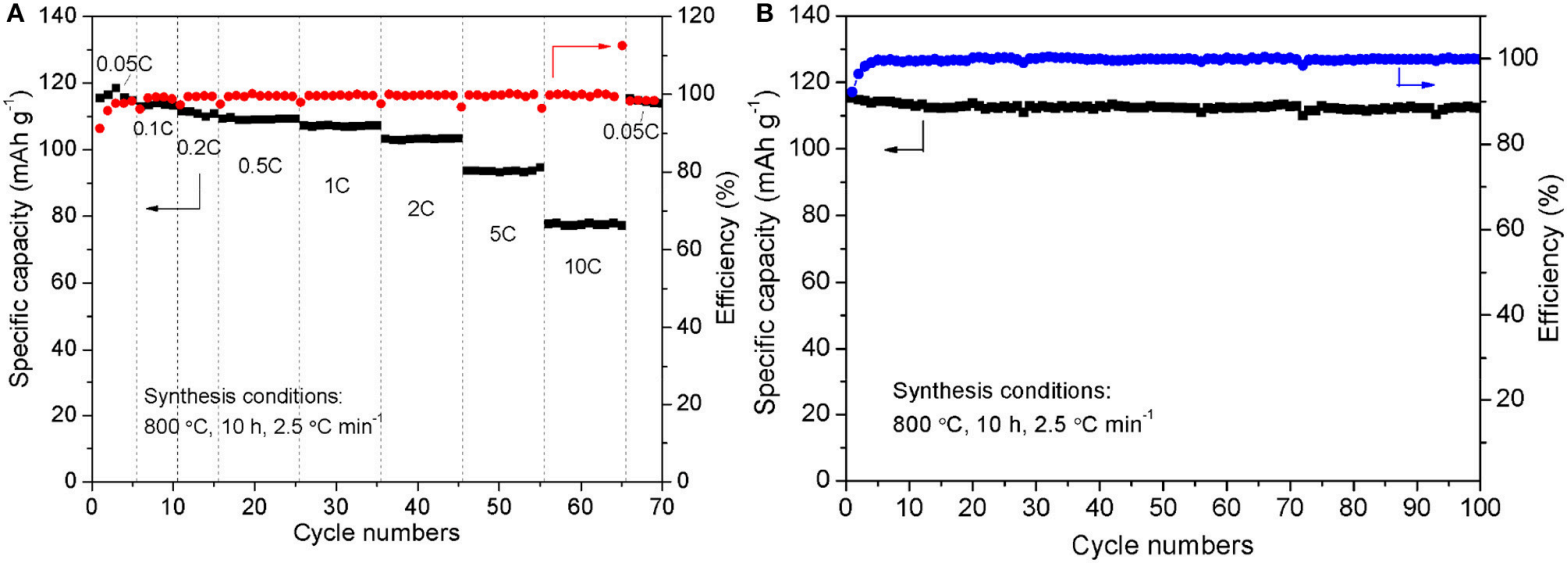
Rate-cycle curves **(A)** and cycling performance at 0.05 C rate **(B)** of the Na_3_V_2_(PO_4_)_3_/C synthesized at the optimal synthesis conditions.

Through the above analysis, the optimal synthesis conditions are as follows: calcine at 800°C for 10 h with a heating rate of 2.5°C min^−1^. For the Na_3_V_2_(PO_4_)_3_/C sample synthesized at the optimal synthesis conditions, the rate-cycle curves and cycling perormance at 0.05 C rate are shown in Figure 10. The optimal sample exhibits excellent rate performance and cycle stability. From Figure 10A, it can be noticed that as the current rate reverses back to 0.05 C after cycling at different C rates, the specific capacity can recover to nearly initial values, indicating the good reversibility and structural stability of the Na_3_V_2_(PO_4_)_3_/C sample. And when 100 times cycle at 0.05 C (Figure 10B), the capacity retention reaches up to 97.0%.

## Conclusions

A series of Na_3_V_2_(PO_4_)_3_/C nanofibers are synthesized through a pre-reduction assisted electrospinning method. Under the different synthesis conditions, the morphology, and electrochemical performance of the Na_3_V_2_(PO_4_)_3_/C nanofibers are obviously different. As the temperature increases, the nanofibers become thinner, but gradually lose the filamentous morphology. With the elongation of the calcining time, shoot-like crystalline particles on the fiber surface can be re-melted into nanowires. The heating rate also play a critical role in the structure and morphology of the Na_3_V_2_(PO_4_)_3_/C samples. As a result, the structure, morphology, and electrochemical performance of Na_3_V_2_(PO_4_)_3_/C composite nanofibers can be controlled by adjusting the heat treatment parameters. Under the optimum synthesis conditions of 800°C, 10 h and heating rate of 2.5°C min^−1^, the obtained Na_3_V_2_(PO_4_)_3_/C composite nanofibers present excellent electrochemical performances.

## Author Contributions

LW, YH, and SS did the main experiment and write the manuscript. XZ and HL envolved the discussion of the experiment and revised the manuscript. LY assisted the material synthesis. YS and SZ made the research plan. SZ and LW also provided the financial support.

### Conflict of Interest Statement

The authors declare that the research was conducted in the absence of any commercial or financial relationships that could be construed as a potential conflict of interest.
